# A Prospective Review of Preoperative Nutritional Status and Its Influence on the Outcome of Abdominal Surgery

**DOI:** 10.7759/cureus.19948

**Published:** 2021-11-27

**Authors:** Bharathi Akula, Nilesh Doctor

**Affiliations:** 1 Surgery, University Hospital of Leicester, Leicestershire, GBR; 2 Gastrointestinal Surgery, Jaslok Hospital and Research Centre, Mumbai, IND

**Keywords:** preoperative nutrition, subjective global assessment, complications, impact on outcome, screening of nutritional status

## Abstract

Aim

To assess the incidence of malnutrition in a surgical gastroenterology unit and analyze its impact on postoperative complication rates.

Method

Data were prospectively accrued from patients admitted for emergency or elective surgery to the gastrointestinal surgery unit at Jaslok Hospital between May 2013 and May 2014. The nutritional status was preoperatively assessed by using anthropometric parameters like body mass index (BMI), midarm circumference (MAC), and tissue skinfold thickness (TSFT). In addition, a subjective global assessment scale (SGA), serum albumin, and absolute lymphocyte count (ALC) were used. Patients with BMI <18.5, MAC <24 cm in males and <22 cm in females, and TSFT <10 mm were considered malnourished. Patients with serum albumin between 3 and 3.5 g/dl were considered mild, 2.4-2.9 g/dl was moderate, and <2.4 g/dl were severely malnourished. Patients with ALC between 1200 and 2000/cm were labelled mild, between 800 and 1199/cm were moderate, and <800/cm were severely malnourished. As per SGA, well-nourished had less than 5% weight loss or if more than 5%, with recent gain and improved appetite, mild/moderately malnourished had 5% to 10% weight loss with no gain, mild subcutaneous fat loss, and those severely malnourished had more than 10% weight loss, severe subcutaneous fat loss, and muscle wasting. Postoperative complications were graded as per the Clavien-Dindo classification. Patients with grades 1 and 2 complications were labelled as minor and the rest as major.

Result

Men in the age group of 40-60 years comprised the majority of the study population. The most frequent reason for admission was cholelithiasis. The overall incidence of malnutrition was 22.16%. Out of the 96 patients who had complications, 45 had minor and 41 had significant complications. Amongst the well-nourished, the incidence of complications was 26.62% of which the majority were minor complications. Severely malnourished patients had a high complication rate (63.38%); 32% out of the 63.38% developed significant complications. The majority of the patients suffering from severe malnutrition belonged to the sub-group with chronic pancreatitis and carcinoma of the pancreas. According to the *chi-square* analysis of the data, ALC, serum albumin, and SGA correlate with the postoperative complication rate with a p-value <0.05 as significant. On the contrary, BMI, MAC, and TSFT did not correlate with postoperative complications.

Conclusion

Preoperative malnutrition is common among patients undergoing abdominal surgeries in the urban private health care sector. Although there have been studies that have analyzed the incidence of malnutrition in patients undergoing oncological surgeries, there is limited literature on malnutrition among patients subjected to gastrointestinal surgeries. We conclude that simple bedside nutritional assessment tools like serum albumin, absolute lymphocyte count, and SGA can accurately identify malnourished patients preoperatively and are good predictors of postoperative complications. Hence, it is imperative to assess and attempt to improve the nutritional status of the patients preoperatively.

## Introduction

Malnutrition is the greatest single threat to the world’s public health. Despite its high prevalence amongst hospitalized patients, it is routinely underdiagnosed and remains unaddressed, especially in developing nations. Worldwide, 20% to 50% of hospitalized patients are malnourished [1]. Malnutrition is associated with tissue and muscle wasting and multiorgan dysfunction, contributing to increased risk of post-surgical morbidity and prolonged duration of stay. Modern research has demonstrated the benefits of screening preoperative nutritional status and appropriate surgical care treatment.

Patients undergoing gastrointestinal surgery suffer from decreased oral intake, tumour cachexia, impaired absorption due to intestinal obstruction, or reduced bowel length affecting their nutritional status. Other surgical parameters like preoperative sepsis, American Society of Anesthesiology (ASA) score of more than 3, emergency surgery, open surgery, long duration of surgery, and massive intraoperative blood loss contribute to the poor postoperative outcome. Moreover, low socioeconomic status, Indian customs, restricting particular food intake pose an additional risk.

Forty per cent to 65% of patients admitted to surgical wards are malnourished [2]. The fact that this percentage seems to be unchanged over time is alarming. Preoperative nutritional screening is not routinely conducted in many Indian tertiary care hospitals. A thorough literature search failed to show any scientific study performed in the past to assess the preoperative nutritional status amongst Indian patients and was, therefore, the main reason for conducting this study.

## Materials and methods

This study included 225 patients admitted to the gastrointestinal surgery department at Jaslok Hospital, Mumbai, India, between May 2013 and December 2014. Patients who explored outside our institute but re-explored at Jaslok Hospital were excluded. Nutritional status was assessed on admission by anthropometric (body mass index (BMI), midarm circumference, triceps skinfold thickness), biochemical (serum albumin, absolute lymphocyte count), and objective tools (SGA: subjective global assessment ). The triceps skinfold thickness was measured using a vernier calliper. SGA was calculated based on recent changes in dietary intake, body weight, gastrointestinal symptoms, and loss of subcutaneous fat and muscle mass.

Those with BMI <18.5, mid-arm circumference (MAC) <24 cm in males and <22 cm in females, and tissue skinfold thickness (TSFT) <10 mm were considered malnourished. Patients with serum albumin between 3 and 3.5 g/dl were considered mildly malnourished while those with 2.4-2.9 g/dl and <2.4 g/dl were labelled as moderately and severely malnourished, respectively. Patients with absolute lymphocyte count (ALC) between 1200 and 2000/mm^3^ were labelled as mild, between 800 and 1199/mm^3^ were grouped as moderately malnourished, and <800/mm^3^ were severely malnourished. As per SGA, well-nourished patients had less than 5% weight loss or if more than 5% with recent gain and improved appetite, mild/moderately malnourished had 5% to 10% weight loss with no gain, mild subcutaneous fat loss, and those who were severely malnourished had more than 10% weight loss, severe subcutaneous fat loss, and muscle wasting.

Comparison between two categorical variables was performed with a chi-square test. The Mann Whitney test was applied to compare each independent variable with complications, as it was much more superior than the t-test to indicate significance. Independent predictors of complication were identified using binary regression analysis. The Clavien-Dindo (C-D) classification was used to grade these complications. The following infectious complication characteristics recorded were: (1) intra-abdominal infection; (2) respiratory infection; (3) wound infection; (4) urinary tract infection; and (5) venous catheter-related infection. Infectious complications were diagnosed based on clinical signs of sepsis, imaging features, raised inflammatory markers, and positive culture reports. The Clavien-Dindo (C-D) classification was adopted to assess these complications.

## Results

The majority of the study population comprised men aged 40 to 60 years, indicating that they are more prone to seek health care than women in India. The most frequent reason for admission was cholelithiasis. Worldwide, gall stone disease is the commonest cause of access to acute surgical wards, as demonstrated in Table 1.

**Table 1:** Distribution of organ diagnoses ANP: acute necrotizing pancreatitis; CR: benign colorectal; SBO: small bowel obstruction; CP: chronic pancreatitis; CRC: colorectal cancer; IBD: inflammatory bowel disease; IsBD: ischemic bowel disease; GB: gallbladder stone disease; UGI: upper gastrointestinal

Organ diagnosis	No.	Percentage
Abdominal Kochs	1	0.4%
ANP	2	0.9%
Appendicitis	13	5.8%
Benign biliary	10	4.4%
Benign CR	14	6.2%
Benign perianal	9	4.0%
Benign SBO	23	10.2%
Ca pancreas	11	4.9%
CP	6	2.7%
CRC	10	4.4%
IBD	2	0.9%
IsBD	3	1.3%
Liver benign	5	2.2%
Malignant biliary	4	1.8%
Malignant CR	1	0.4%
Malignant GB	1	0.4%
Malignant liver	12	5.3%
Others	24	10.7%
Pancreas others	9	4.0%
Symptomatic gallstones	48	21.3%
UGI benign	10	4.4%
UGI malignant	7	3.1%
Total	225	100.0%

The prevalence of malnutrition measured by various nutritional assessment tools ranged from 4% to 35%, as shown in Tables 2-7. This wide variation was attributed to the fact that BMI is particular and serum (Sr.) albumin is highly sensitive.

**Table 2:** Prevalence of malnutrition by BMI BMI: body mass index

BMI	No.	Percentage
Underweight	9	4.0%
Normal	89	39.6%
Overweight	86	38.2%
Obese	41	18.2%
Total	225	100.0%

**Table 3:** Prevalence of malnutrition by TSFT TSFT: tissue skinfold thickness

TSFT status (>13 cm = Normal)	No.	Percentage
Malnourished	96	42.7%
Normal	129	57.3%
Total	225	100.0%

**Table 4:** Prevalence of malnutrition by MAC MAC: mid-arm circumference

MAC status (> 26= Normal)	No.	Percentage
Malnourished	136	60.4%
Normal	89	39.6%
Total	225	100.0%

**Table 5:** Prevalence of malnutrition as per serum (Sr) albumin levels

Sr. Albumin	No.	Percentage
Severe	10	4.4%
Moderate	15	6.7%
Mild	55	24.4%
Normal	145	64.4%
Total	225	100.0%

**Table 6:** Prevalence of malnutrition as per serum (Sr) creatinine

Sr. Creatinine	No.	Percentage
Malnourished	28	12.4%
Normally nourished	197	87.6%
Total	225	100.0%

**Table 7:** Prevalence of malnutrition by SGA SGA: subjective global assessment

Nutritional status by SGA	No.	Percentage
Severely malnourished	13	5.8%
Moderately malnourished	16	7.1%
Mildly malnourished	42	18.7%
Well nourished	154	68.4%
Total	225	100.0%

Sixty-one point eight per cent (61.8%) had no complications, 18.2% had significant complications, 5.4% were reoperated, and the mortality rate was 3.6%. Clavien-Dindo grade 1 was the most common complication, as illustrated in Table 8.

**Table 8:** Incidence of various complications

Grade of complication	No.	Percentage
0	139	61.8%
1	44	19.6%
2	1	0.4%
3	4	1.8%
3a	12	5.3%
3b	8	3.6%
4a	3	1.3%
4b	6	2.7%
5	8	3.6%
Total	225	100.0%

In this study, SGA and Sr. albumin correlate significantly with postoperative complications by univariate analysis depicted in Table 9. MAC and ALC did not correlate with complications directly. An increasing trend of the incidence of complications was seen with an increase in the severity of malnutrition, whereas when the Mann-Whitney test was applied, absolute lymphocyte count <2300, serum albumin <3.5, Hb <11, ASA score>2, packed cell volume (PCV) <32, and age >53 correlated with postoperative complications.

**Table 9:** Association between malnutrition assessment index and postoperative complications BMI: body mass index; SGA: subjective global assessment; TSFT: tissue skinfold thickness

Variables	B	S.E.	Wald	df	Sig.	Exp(B)
BMI (Underweight (< 18.5)	-0.845	1.060	0.635	1	0.425	0.430
Sr. Albumin (Abnormal)	0.129	0.378	0.117	1	0.733	1.138
SGA (Malnourished)	1.699	0.383	19.707	1	9.03E-06	5.467
TSFT (Malnourished)	0.081	0.854	0.009	1	0.924	1.084
Mid-Arm Circumference (Malnourished)	-1.032	0.679	2.312	1	0.128	0.356
Sr. Creatinine (Malnourished)	0.209	0.460	0.207	1	0.649	1.233
Constant	-1.004	0.206	23.69	1	0.000	0.366

Severely malnourished patients had a higher complication rate (63.38%) than those who were well-nourished (26.62%). Also, the severely malnourished were found to develop significant complications (32% out of the 63.38%). The most typical major complication is intraabdominal sepsis treated either by image-guided drainage or surgical re-exploration. Fifteen per cent (15%) developed a super skin infection. The rate of non-infectious postoperative complications was nearly similar amongst the well-nourished and severely malnourished (8.8% versus 6.4% ). Mortality amongst the malnourished was 4.6% versus 0.4% in the well-nourished.

The incidence of minor and major complications identified by Clavin-Dindo classification amongst patients undergoing primary and minor procedures is shown in Table 10.

**Table 10:** Complication rates

Degree of complication	No.	Percentage
Major complication	41	18.2%
Minor complication	45	20.0%
No complication	139	61.8%
Total	225	100.0%

## Discussion

Malnutrition is associated with complications like a high risk of infection, prolonged duration of stay, and increased mortality. This study prospectively evaluated the nutritional status of 225 patients undergoing gastrointestinal surgery on admission and analyzed its impact on postoperative complications. BMI is one of the commonly used indices to assess nutritional status in the hospital. BMI seems to underestimate malnutrition due to its high specificity. Garth AK et al. evaluated the nutritional status of patients undergoing a major oncological procedure for various gastrointestinal cancer using BMI [3]. Their studies failed to identify any correlation between BMI and postoperative complications. Thomas EJ et al. also confirmed that BMI does not significantly correlate with postoperative complications or the length of hospital stay [4]. However, their study demonstrated that overweight patients who underwent abdominal procedures had higher wound infection rates. House MG et al., who tried to identify the preoperative predictors for complications after pancreaticoduodenectomy, found that those with BMI >30 kg/m^2^ had an increased risk of postoperative pancreatic fistulas and wound infection compared to the nonobese patients [5]. The current study found no significant correlation between BMI and postoperative complications, but the overall incidence of complications was high among those overweight (44.2%) as compared to those who were normal ( 38.2%) or underweight (22.2%).

In this study, receiver operating characteristic (ROC) curve analysis was used to determine the optimal cut-off values of tissue skin fold skinfold thickness and mid-upper arm circumference to identify the prevalence of malnutrition. There are no internationally accepted cut-off values to determine malnutrition by MAC and TSFT. Ravasco P et al., in their study based on the nutritional status assessment of critical patients in the ICU, suggested that mid-upper arm circumference (MUAC) is the most straightforward and feasible nutritional assessment tool and, if classified by percentiles, may prove to have prognostic value in identifying those at nutritional risk [6]. James WP et al. suggested that the combined use of BMI and MUAC was a better indicator of identifying patients at nutritional risk and found that MUAC, when used individually, did not correlate with postoperative complications [7, 8].

In a prospective study carried on 99 patients over 18 years of age submitted to elective abdominal surgery in Brazil, malnutrition percentages were similar to those found in this study by MAC, TSFT, and BMI. Their analysis highlights that no method used to assess preoperative nutritional status is free from error and that a combination of different techniques helps diminish the probability of error caused by the isolated use of any one of them. However, in a retrospective analysis of 215 patients submitted to gastrointestinal and abdominal wall surgeries, malnutrition was diagnosed by MAUC and TSFT (both standardized for sex and age, and the percentage of adequacy was calculated [9-10]. Nutritional status assessed by MAC and TSFT did not correlate with postoperative complications. Similarly, this study also demonstrated no correlation between MUAC and TSFT and postoperative complications either with univariate or multivariate analysis.

This study found that serum albumin played a significant role in assessing nutritional status and correlated with postoperative complications indirectly. Weinsier constructed a likelihood of malnutrition score (LOM) score based on the following: body weight, tricep skinfold thickness, and mid-arm circumference, serum folate, vitamin C and serum albumin, and absolute lymphocyte count and hematocrit [11]. This score suggested that 48% of patients were malnourished on admission by LOM score, which showed an association with more extended hospitalization and increased mortality. The Prognostic Nutritional Index, developed by Muller and Buzzy and their associates, includes serum albumin, serum transferrin, and TSFT to assess the nutritional status [12-13]. Lohsiriwat V et al. identified hypo-albuminemia as a significant risk factor for postoperative complications following rectal cancer surgery [14]. This study found similar results; patients with hypoalbuminemia had a higher complication rate (50%) as compared to those with normal albumin (37%).

Takaya K et al. found that preoperative low absolute lymphocyte count was a strong predictor of high mortality and morbidity amongst patients undergoing esophagectomy, suggesting that absolute lymphocyte count has a better predictive value of the nutritional status among cancer patients than in general [15]. There was no statistically significant correlation between absolute lymphocyte count and postoperative complications; however, an increasing trend of complications was seen as the severity of malnutrition increased. Therefore, when the Mann-Whitney test was applied, absolute lymphocyte count correlated with postoperative complications. Kusne S et al. analyzed in 101 consecutive liver transplant patients that preoperative T helper lymphocytes to T suppressor lymphocytes ratio <2.8 in the pretransplant period was associated with severe viral and fungal infections [16]. Gomez D et al. found that preoperative neutrophil to lymphocyte ratio > 5 was an adverse predictor of disease-free and overall survival after curative resection for HCC [17]. Albaran RG et al. retrospectively identified that CD4 + T lymphocyte count <200 correlated with increased postoperative morbidity and mortality among 443 HIV-infected patients undergoing major abdominal surgery [18].

In 1984, Detsky AS evaluated the accuracy of nutritional assessment techniques applied to hospitalized patients and found that SGA has the highest sensitivity and specificity, i.e., 0.82 and 0.72, respectively. The second best nutritional assessment indices were prognostic nutritional index (PNI) with a sensitivity of 0.88 and specificity of 0.45 [19]. According to this study, 31.6% were malnourished by SGA, similar to the IBRANUTRI study, which showed 39% was the incidence of malnutrition assessed by SGA amongst the surgical patients in Brazil [20]. Ryu SW et al. observed that 31% of the patients with gastric cancer submitted to surgery were malnourished by SGA [21]. Thieme RD et al., however, found that 65.6% of patients undergoing abdominal surgery were malnourished and preoperative SGA did not show a positive correlation with postoperative complication [22]. SGA was used to screen 438 patients in Vietnam, out of which 274 patients undergoing major abdominal surgery and found that 42.3% were malnourished with an increased incidence of complication was higher in SGA class C.(33.6% versus 6% in class A) [23]. It was found that SGA has a higher positive predictive value than NRI in predicting the outcome after abdominal surgery (80% versus 74%) [24]. Both hypo-albuminemia and SGA are independent predictors of postoperative complications, which was similar to the results found in this study. Therefore, it is proven beyond doubt by several studies that SGA is identified as the gold standard test to determine patients with malnutrition.

In a similar perspective prospective cross-sectional study conducted by Siribumrungwong B et al. to determine the prevalence of malnutrition amongst 106 patients undergoing abdominal surgeries, 27% were malnourished (nutritional status assessed by European Society for Clinical Nutrition and Metabolism (ESPEN) criteria) [25-27]. Their study also found that malnutrition was significantly associated with postoperative complications after adjusting for other confounding variables with an odds ratio of 3 and 95% CI of 1.1 and 8.4. In Kenya, Dr Beatric Nyanchama at Kenyatta University in 2011 studied the effects of the nutritional status on surgical outcome amongst patients before undergoing abdominal surgery. He identified that 50% were malnourished by SGA on admission, and this prevalence further increased by 16% after surgery. They also demonstrated a significant association between nutritional status and postoperative complications.

Malnourished patients must be identified promptly on admission to prevent adverse postoperative outcomes. Routine nutrition screening using one of the internationally validated nutrition screening indices like NRI or subjective parameters like SGA could provide a basis for dietetic referrals leading to the prescription of appropriate nutrition support [28]. Perioperative malnutrition is a modifiable and treatable cause of perioperative morbidity [29]. The risk of infection, complication, length of hospital stay, and cost are reduced by perioperative nutritional support.

A Cochrane review published in 2008 reviewed 36 studies to examine the impact of dietary advice and supplements in adults with illness‐related malnutrition. The authors concluded that dietary advice plus nutritional supplements might be more effective than advice alone or no advice on measuring short-term weight gain. However, they highlighted the lack of beneficial evidence in managing illness-related malnutrition. In our study, malnourished patients were started on enteral nutritional support within 24 hours. At the same time, those severely malnourished were started on parenteral nutrition to supplement the enteral feeds preoperatively and continued postoperatively until daily calorie intake was reestablished through a regular oral diet.

A randomized trial conducted by the Veterans Affairs Total Parenteral Nutrition Cooperative Study Group [30] found a significant reduction in postoperative complications amongst patients who received parenteral nutrition perioperatively. Patients at risk of malnutrition or identified to be malnourished should receive early dietary care. According to this study, the prevalence of malnutrition assessed by BMI, TSFT, MAC, Sr. albumin, ALC, and SGA was 4%, 4.9%, 10.2%, 36.6%, 34%, 31.6%, respectively. Postoperative complications are significantly correlated with malnutrition estimated by SGA with an odds ratio of 0.499 and a 95% confidence interval limit of 0.091 to 2.213. Even though the results of this study seem comparable with the literature, there are certain shortcomings in this study. The sample size is comparatively smaller and includes benign and malignant cases. This study was conducted in a single centre; hence, results cannot be generalized. Emergency and elective surgeries need to be analyzed separately in future studies.

## Conclusions

Malnutrition persists to be a significant burden on health care facilities worldwide. Preoperative malnutrition is a modifiable and treatable cause of perioperative morbidity despite ignoring early identification. The impact of preoperative nutritional status on postoperative outcomes has to be acknowledged by surgeons and hospitals across the globe. This study suggests that the nutritional status of all patients be assessed on admission and preferably made mandatory by health care organizations. In order to accurately identify the prevalence of malnutrition, a validated nutritional assessment tool, such as SGA, could be used. Therefore, we recommend screening for malnutrition on admission and drafting a nutrition care pathway to prevent adverse postoperative outcomes.
